# Clinical characteristics of cadonilimab-associated infusion reactions: a severity-stratified analysis of 52 cases and preliminary management framework

**DOI:** 10.3389/fimmu.2026.1815365

**Published:** 2026-10-09

**Authors:** Jinlan Li, Rui Qiu, Guisen Yin, Yong Pan, Po Chen

**Affiliations:** 1 Department of Pharmacy, Hunan Cancer Hospital, The Affiliated Cancer Hospital of Xiangya School of Medicine, Central South University, Changsha, Hunan, China; 2 Department of Pharmacy, The People’s Hospital of Jianyang City, Chengdu, Sichuan, China; 3 Department of Clinical Pharmacy, Yantai Traditional Chinese Medicine Hospital, Yantai, Shandong, China; 4 Department of Gastroenterology and Urology, Hunan Cancer Hospital, The Affiliated Cancer Hospital of Xiangya School of Medicine, Central South University, Changsha, Hunan, China

**Keywords:** bispecific antibody, cadonilimab, clinical features, infusion-related reaction, preliminary management framework

## Abstract

**Objective:**

To describe the clinical characteristics of infusion-related reactions (IRRs) associated with cadonilimab in a real-world cohort and propose preliminary management considerations based on these observations.

**Methods:**

A descriptive retrospective case series was conducted, involving data collection from 52 patients who received cadonilimab and subsequently experienced infusion-related reactions at a tertiary hospital between July 2022 and December 2025. Patients were classified into a common IRR group (n=31) and a severe IRR group (n=21) based on the severity criteria outlined in CTCAE 6.0. We conducted a comparative analysis of baseline characteristics, IRR onset timing, clinical manifestations, management strategies, and outcomes between the two groups.

**Results:**

The median time to IRR onset was 20 minutes post-infusion, with the highest proportion of recorded IRR cases occurring during the second treatment cycle (42.3%). Severe IRR manifested as multi-organ involvement in 61.9% of cases, predominantly affecting the respiratory and cardiovascular systems. Patients with severe IRR more frequently received corticosteroids combined with antihistamines (57.1% vs. 29.0%) and had higher rates of premedication (76.2% vs. 45.2%). Twenty-four patients (46.2%) attempted same-cycle infusion resumption; among these, 15 (62.5%) successfully completed the infusion without symptom recurrence. Among the 10 severe-group patients who attempted same-cycle infusion resumption, 6 (60.0%) experienced recurrent symptoms; among all 21 severe-group patients, the recurrence rate was 28.6% (6/21). The clinically relevant same-cycle recurrence rates were 9/24 overall (37.5%), 3/14 (21.4%) in the common group, and 6/10 (60.0%) in the severe group.

**Conclusion:**

Cadonilimab is associated with early-onset infusion-related reactions frequently involving multiple organ systems. Based on our institutional experience, we propose a preliminary management framework that requires prospective validation before it can be recommended for routine clinical practice.

## Introduction

Cadonilimab, a bispecific antibody simultaneously targeting PD-1 and CTLA-4, has demonstrated considerable therapeutic efficacy against various advanced solid tumors through its dual immune checkpoint blockade (1, 2). However, its potent immune-activating properties are associated with a distinct safety profile, among which infusion-related reactions (IRRs) rank among the most frequently observed adverse events and require prompt clinical management (1). Clinically, IRRs exhibit a heterogeneous spectrum of manifestations, ranging from mild cutaneous symptoms, such as flushing and pruritus, to severe and potentially life-threatening events, including respiratory distress and circulatory failure (3). The underlying mechanisms may involve diverse pathophysiological processes, notably cytokine release and hypersensitivity/allergic responses.

To date, although management guidelines for infusion-related reactions associated with conventional chemotherapy and monoclonal antibodies have been established (4), evidence regarding optimal management strategies for bispecific antibodies, including cadonilimab, remains limited—particularly that derived from real-world clinical practice. Consequently, clinical management of these reactions often relies on empirical approaches, resulting in considerable heterogeneity in premedication protocols, acute interventions, and rechallenge decision-making. Such inconsistency may compromise patient safety and treatment continuity.

A recent case series by Liang et al. (5) reported 7 cases of cadonilimab-associated IRRs, describing their basic clinical manifestations and management. While informative, that study was limited by its small sample size and descriptive nature, precluding comparative analyses across severity grades and any systematic evaluation of rechallenge outcomes. The present study extends this literature in four important respects: (1) sample size – with 52 cases, it represents the largest real-world cohort of cadonilimab-associated IRRs reported to date (2); severity-stratified analysis – we systematically compared the clinical features, management, and outcomes of grade 1–2 versus grade 3–5 IRRs (3); rechallenge evaluation – we separately analyzed same-cycle infusion resumption and re-exposure in subsequent cycles, providing detailed recurrence-rate data; and (4) exploratory risk-signal identification – we identified potential associations that may inform future hypothesis-driven studies. None of these analytical dimensions were feasible in the previously published 7-case series.

Therefore, an urgent need exists to characterize the specific clinical features of cadonilimab-associated IRRs, so as to inform the development of preliminary management recommendations. To this end, this study retrospectively analyzed clinical data from 52 cases of Cadonilimab-related IRRs to delineate their clinical features. Drawing on these findings and established IRR management principles, we further propose, based on our institutional experience, a preliminary management framework, which requires prospective validation.

## Materials and methods

### 1 Study design and data source

We conducted a single-center retrospective case series based on data extracted from the electronic medical records of a tertiary care hospital. Consecutive patients who received cadonilimab, either as monotherapy or in combination regimens, between July 2022 and December 2025 and developed documented adverse drug reactions (ADRs) were included. Cases of IRRs were identified through a systematic review of ADR reports and the corresponding medical records. An IRR was defined as an acute adverse event occurring during or within 24 hours after cadonilimab infusion.

### 2 Inclusion and exclusion criteria

Cases were included if they met all of the following criteria (1): age ≥18 years (2); histologically confirmed malignancy (3); treatment with cadonilimab, either as monotherapy or in combination with other agents; and (4) documented IRR (as defined above) with complete clinical records.

Cases were excluded if they met any of the following criteria (1): IRR symptoms attributable to concomitant medications (e.g., chemotherapeutic agents) or alternative etiologies (e.g., infection or tumor progression); or (2) insufficient clinical data precluding IRR evaluation.

### 3 Grouping and evaluation criteria

IRRs were graded according to the Common Terminology Criteria for Adverse Events (CTCAE) Version 6.0 (6), and categorized for analysis as follows: grade 1–2 reactions were designated the “Common IRR group”, and grade 3–5 reactions the “Severe IRR group”. All subsequent analyses compared these two groups.

The patient served as the unit of analysis. For patients who experienced IRRs in multiple treatment cycles, the index IRR was defined as the most severe IRR episode; in cases of equal severity, the earliest event was selected. All analyses – including severity grouping, onset timing, clinical manifestations, management strategies, and recovery time – were based on this index event. Multisystem involvement was assessed according to the organ systems affected during the index event rather than cumulatively across cycles.

Causality between cadonilimab and each observed IRR was independently assessed by two clinical pharmacists according to the China Adverse Drug Reaction Reporting and Monitoring Guidelines (7), based on the following considerations (1): the temporal relationship between IRR onset and cadonilimab infusion (within 24 hours) (2); the consistency of manifestations with the known clinical characteristics of cadonilimab-associated IRRs (3); symptom resolution after drug withdrawal or symptomatic treatment (4); the exclusion of alternative etiologies, including concomitant chemotherapy or targeted agents, infection, and tumor progression; and (5) recurrence upon re-exposure, when applicable. Causality was classified as ‘certain,’ ‘probable/likely,’ ‘possible,’ ‘unlikely,’ or ‘unevaluable.’ Disagreements between the two assessors were resolved through discussion. The detailed causality assessments are provided in **Supplementary Table S1**.

### 4 Data collection and processing

Data were collected on the following domains (1): baseline patient characteristics, including age, sex, tumor type, disease stage, and treatment regimen (2); IRR-related parameters, including onset cycle, time to onset relative to infusion, affected organ systems, and clinical manifestations (3); management strategies, including premedication, acute pharmacological interventions (e.g., corticosteroids and antihistamines), and supportive measures (e.g., oxygen therapy); and (4) clinical outcomes, including time to symptom resolution, rechallenge status, and outcomes in subsequent treatment cycles.

Continuous variables are expressed as mean (standard deviation) or median (interquartile range [IQR], range), as appropriate. Categorical variables are presented as frequencies and percentages. Between-group comparisons of continuous variables were performed using the Wilcoxon rank-sum test (for two groups) or the Kruskal-Wallis test (for three or more groups). Categorical variables were compared using the chi-square test, unless any expected cell frequency was <5, in which case Fisher’s exact test was applied.

Covariate balance was assessed using standardized mean differences (SMDs), with SMD< 0.1 considered indicative of negligible imbalance. All statistical analyses were performed using R software (version 4.2.2) and MSTATA (http://www.mstata.com) (8). Two-sided P-values < 0.05 were considered statistically significant.

### 5 Methodology for preliminary management framework development

Based on our findings and established IRR management principles (3, 4), we developed a preliminary management framework through the following steps (1): extracting the preventive and therapeutic measures that used in this cohort (2); defining critical decision points for severity-stratified care (e.g., criteria for infusion discontinuation and timing of rechallenge); and (3) integrating these components into a structured clinical pathway. This framework is hypothesis-generating and requires prospective validation.

## Results

### 1 Patient baseline characteristics

A total of 52 patients who developed IRRs were included in the analysis. The patient disposition flow diagram is shown in **Figure 1**. Of these patients, 21(40.4%) were assigned to the severe IRR group and 31 (59.6%) to the common IRR group. As shown in **Supplementary Table S2**, baseline characteristics showed some imbalances between the two groups. Patients in the severe group were younger than those in the common group (mean ± SD: 51 ± 9 vs. 57 ± 11 years; SMD: 0.54; 95% CI: -0.03 to 1.10). Sex distribution was well balanced between the groups (SMD: 0.05; 95% CI: -0.51 to 0.60).

**Figure 1:**
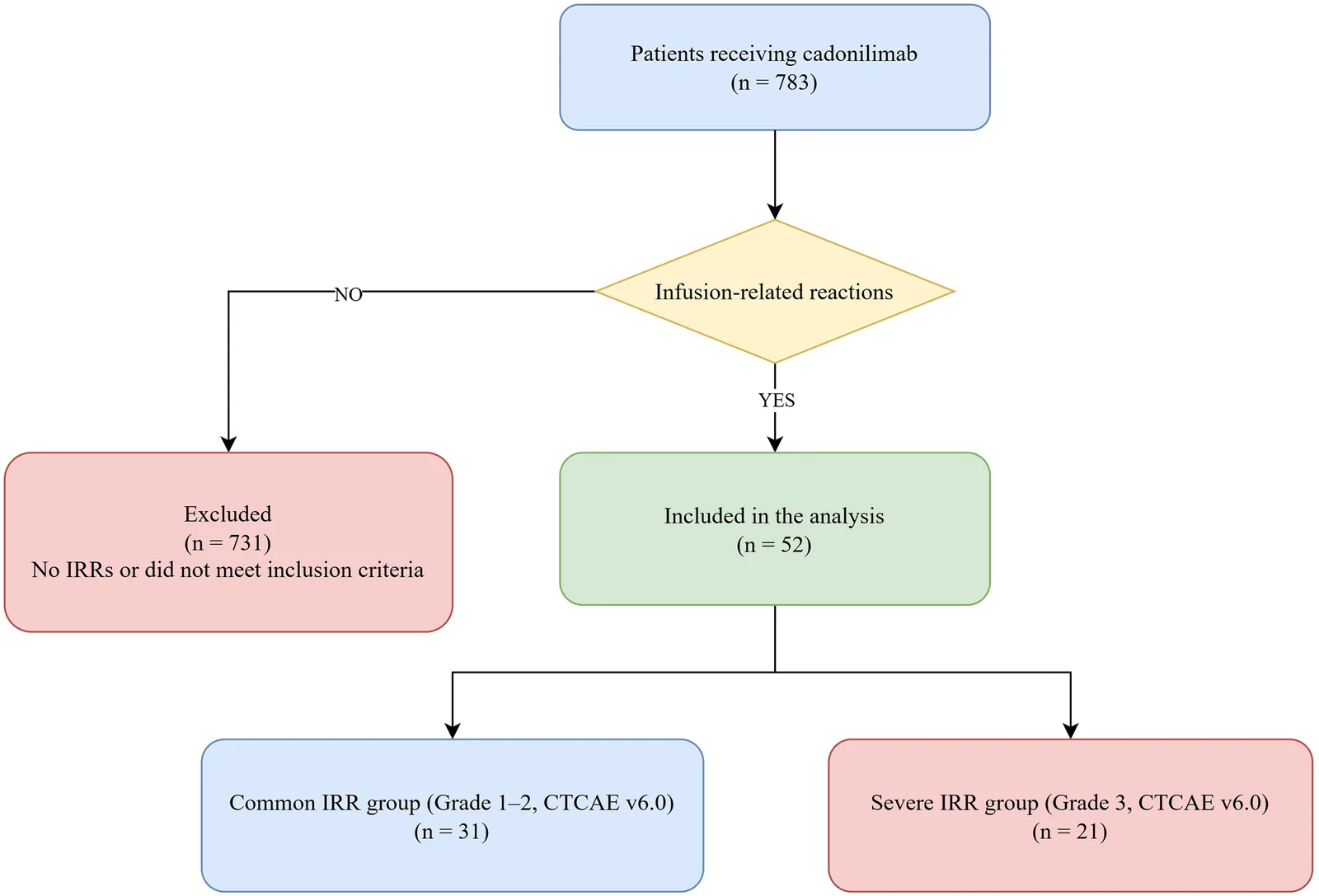
Patient disposition flow diagram. Of 783 patients who received cadonilimab between July 2022 and December 2025 at a tertiary care center, 52 developed infusion-related reactions (IRRs) and were included in the analysis. Patients were stratified into a common IRR group (CTCAE grade 1-2, n = 31) and a severe IRR group (CTCAE grade 3, n = 21).

Regarding tumor characteristics, the distribution of malignancy types differed between the two groups (SMD: 0.71; 95% CI: 0.14 to 1.28), with a higher prevalence of lung cancer in the severe group (33.3% vs. 22.6%). Additionally, the severe group had a higher proportion of patients with stage IV disease (76.2% vs. 54.8%), although this difference was not statistically significant (SMD: 0.46; 95% CI: -0.10 to 1.02).

Regarding treatment characteristics, patients in the severe group more frequently received targeted therapy plus immunotherapy (42.9% vs. 19.4%), whereas those in the common group were more likely to receive chemotherapy plus immunotherapy (61.3% vs. 28.6%). The distribution of combination regimens differed significantly between the two groups(SMD: 0.91; 95% CI: 0.33 to 1.49). Additionally, the severe group had higher rates of third-line or subsequent therapy (33.3% vs. 9.7%; SMD: 0.60; 95% CI: 0.04 to 1.17) and prior exposure to PD-1 inhibitors (42.9% vs. 12.9%; SMD: 0.71; 95% CI: 0.14 to 1.28).

All IRRs occurred during the infusion or shortly after its completion, with a median onset of 20 minutes from the start of infusion. There were no significant between-group differences in time to onset (SMD: -0.26; 95% CI: -0.82 to 0.30) or in the administered dose at the time of onset (SMD: 0.03; 95% CI: -0.52 to 0.59). However, a trend toward a between-group difference was observed in the treatment cycle at onset(SMD: 0.60; 95% CI: 0.03 to 1.16), with the second cycle (C2) accounting for the largest proportion of cases (42.3%).

According to the CTCAE 6.0 criteria, the index IRRs were distributed as follows: grade 1 in 8 cases (15.4%), grade 2 in 23 cases (44.2%), and grade 3 in 21 cases (40.4%); no grade 4 or grade 5 events were observed. Based on these grades, patients were classified into a common IRR group(grade 1–2; n=31) and a severe IRR group(grade 3; n=21).

### 2 Differences in clinical manifestations based on IRR severity

IRRs involved multiple organ systems, with cutaneous (67.3%) and respiratory (51.9%) manifestations being the most common overall. Severe IRRs were significantly more likely to involve multiple systems than common IRRs (61.9% vs. 32.3%). Notably, compared with common IRRs, severe IRRs presented with significantly higher incidences of respiratory symptoms (particularly dyspnea) and cardiovascular manifestations (including chest tightness, palpitations and hemodynamic instability). Systemic symptoms such as chills and fever were common in both groups. Detailed findings are presented in **Supplementary Table S3**, and the symptom distribution by organ system is provided in **Supplementary Table S4**.

### 3 Clinical management measures and outcomes

Regarding acute management, patients with severe IRRs were more likely to receive combination therapy with corticosteroids and antihistamines (57.1% vs. 29.0%). Oxygen therapy was frequently used as supportive care, with an overall rate of 69.2%. The median time to symptom resolution was 43 minutes, with no significant between-group difference (**Table 1**).

**Table 1:** Interventions and outcomes.

Item	N=52	Common, N = 31	Severe, N = 21	P-value
**Drug treatment, n (%)**				0.146^2^
Steroids	20 (38.5%)	14 (45.2%)	6 (28.6%)	
Antihistamines	6 (11.5%)	5 (16.1%)	1 (4.8%)	
Glucocorticoids + Antihistamines	21 (40.4%)	9 (29.0%)	12 (57.1%)	
Fluid Therapy	3 (5.8%)	1 (3.2%)	2 (9.5%)	
Symptomatic treatment	2 (3.8%)	2 (6.5%)	0 (0.0%)	
**Oxygen therapy, n (%)**	36 (69.2%)	20 (64.5%)	16 (76.2%)	0.371^3^
**Recovery time (min)**				0.084^1^
Mean ± standard deviation	70 ± 95	72 ± 55	68 ± 137	
Median (Q1, Q3)	43 (30, 60)	60 (30, 120)	30 (30, 60)	
Min, Max	10, 660	10, 240	10, 660	

^1^ Wilcoxon rank sum test; ^2^Fisher’s exact test; ^3^Pearson’s Chi-squared test. Bold indicates category/item headings and does not denote statistical significance.

### 4 Rechallenge treatment outcomes

Same-cycle infusion resumption: After symptom improvement, 24 patients (46.2%) attempted to resume the ongoing infusion at a reduced rate. Of these, 15 (62.5% of those who attempted resumption, 28.8% of all patients) successfully completed the infusion without symptom recurrence, whereas 9 (37.5% of those who attempted resumption) developed recurrent symptoms during the resumed infusion. The clinically relevant same-cycle recurrence rates were 9/24 overall (37.5%), 3/14 (21.4%) in the common group, and 6/10 (60.0%) in the severe group. The results are summarized in **Table 2A**.

**Table 2A:** Same-cycle infusion resumption.

Outcome	N=52	Common, N = 31	Severe, N = 21	P-value^2^
Resumption attempted, recurrence occurred	9 (17.3%)	3 (9.7%)	6 (28.6%)	0.191
Resumption attempted, completed without recurrence	15 (28.8%)	11 (35.5%)	4 (19.0%)	
No Resumption attempted	28 (53.8%)	17 (54.8%)	11 (52.4%)	
Total resumption attempts	24 (46.2%)	14 (45.2%)	10 (47.6%)	

^2^ Fisher’s exact test.

Subsequent-cycle re-exposure: Among all 52 patients, 22 (42.3%) received cadonilimab in a subsequent treatment cycle after the index IRR. Of these, 8 (36.4% of those re-exposed; 15.4% of all patients) experienced no recurrence, whereas 14 (63.6% of those re-exposed) developed recurrent IRRs. These results are summarized in **Table 2B**. The overlap between the 24 patients who underwent same-cycle resumption and the 22 patients re-exposed in a subsequent cycle is detailed in **Supplementary Table S5**.

**Table 2B:** Re-exposure in subsequent-cycle.

Outcome	N=52	Common, N = 31	Severe, N = 21	P-value^2^
Not re-exposed	30 (57.7%)	18 (58.1%)	12 (57.1%)	0.581^2^
Re-exposed, recurrence occurred	14 (26.9%)	7 (22.6%)	7 (33.3%)	
Re-exposed, no recurrence	8 (15.4%)	6 (19.4%)	2 (9.5%)	
Total re-exposed patients	22 (42.3%)	13 (41.9%)	9 (42.9%)	

^2^ Fisher’s exact test.

Following rechallenge with modified infusion rates, recurrent IRRs occurred in 21.4% (3/14) of patients in the common cohort and 60.0% (6/10) of those in the severe cohort. Among all 52 patients who experienced at least one IRR, the overall same-cycle recurrence rate was 17.3% (9/52): 9.7% (3/31) in the common group and 28.6% (6/21) in the severe group.

### 5 Preliminary management framework based on institutional experience

Based on these findings, we propose a preliminary management framework for cadonilimab-related IRRs (**Figure 2**). This framework provides structured guidance on vigilant monitoring during infusion, early recognition of IRRs, and immediate intervention in accordance with institutional protocols. It represents a preliminary synthesis of our institutional experience and existing guidelines, and its efficacy requires prospective validation.

**Figure 2:**
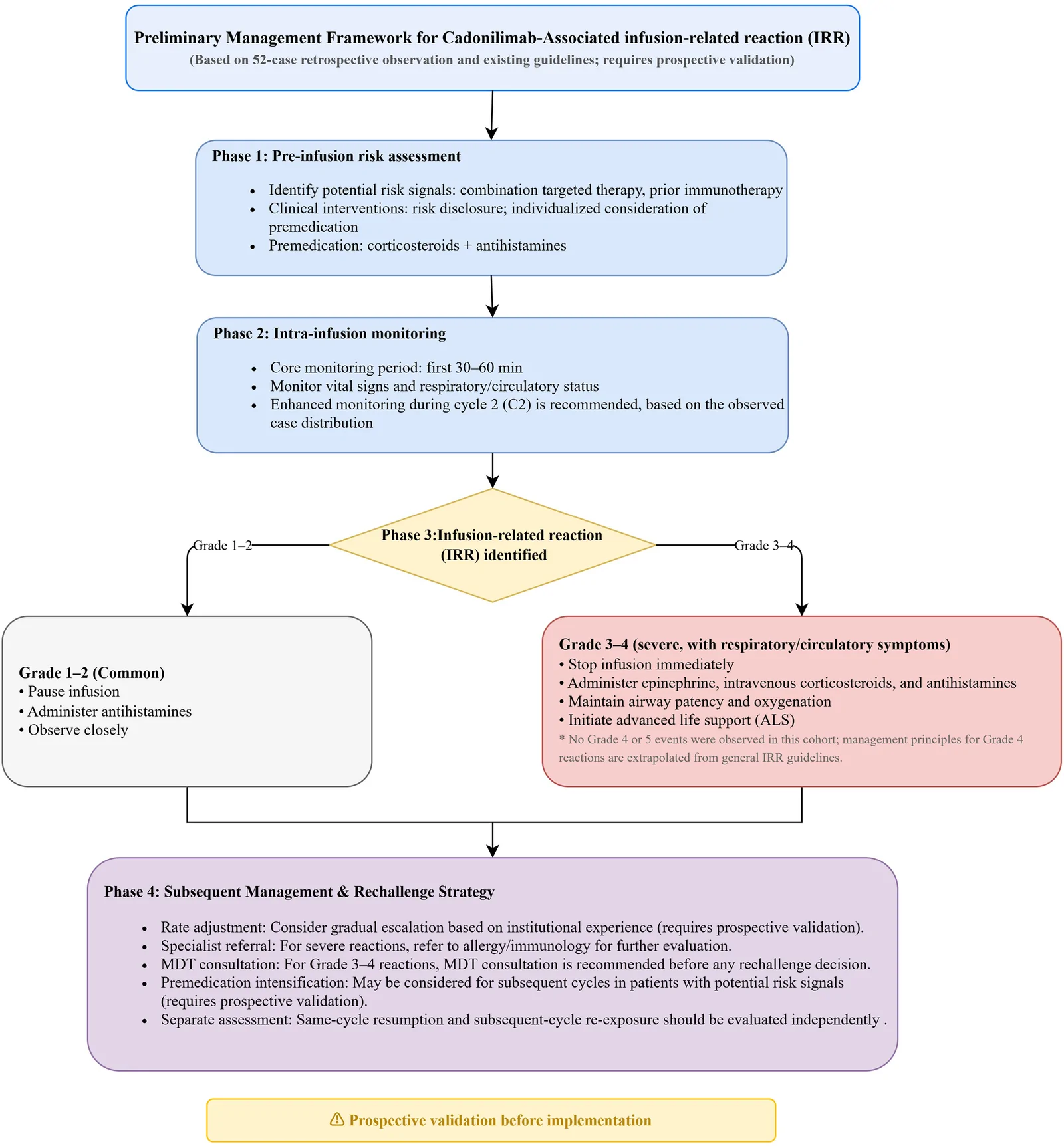
Proposed preliminary management framework for cadonilimab-associated infusion-related reactions derived from a retrospective analysis of 52 cases and current guidelines (pending prospective validation). The framework comprises four components: pre-infusion risk assessment, monitoring during infusion, severity-graded management for grade 1–2 and grade 3–4 infusion-related reactions (IRRs), and subsequent rechallenge strategies. C2, cycle 2; IRR, infusion-related reaction; MDT, multidisciplinary team; ALS, advanced life support.

## Discussion

Through an analysis of 52 real-world cases, this study characterizes the clinical features of cadonilimab-related IRRs and proposes a preliminary management framework. We subsequently discuss the underlying mechanisms, clinical management challenges, the rationale for the proposed framework, and future directions.

### 1 Mechanisms and characteristics of cadonilimab-related IRRs

The median time to IRR onset was 20 minutes after the start of infusion—considerably earlier than the 30–120 minutes typically reported for conventional monoclonal antibodies such as rituximab (9). This temporal profile suggests two potential mechanisms: IgE-mediated hypersensitivity or non-immune-mediated cytokine release associated with target engagement (4, 10). In our cohort, the second treatment cycle (C2) accounted for the largest proportion of IRR events. However, because the denominator population (i.e., the total number of patients receiving cadonilimab in each cycle) was unavailable, this observation does not establish that IRR incidence peaks at C2; rather, it supports enhanced monitoring during C2 infusions, pending confirmation in studies with complete denominator data. This pattern is compatible with, but does not prove, immune sensitization following initial exposure (11).

We hypothesize that the bispecific targeting of PD-1 and CTLA-4 by cadonilimab may potentiate T-cell activation, thereby increasing the risk of cytokine release syndrome (CRS) (12); however our data cannot confirm the specific mechanism. Comparable toxicity profiles have been reported for other bispecific antibodies and combination regimens, such as ipilimumab plus nivolumab, in which severe immune-related adverse events (irAEs) occur in 20–30% of patients (13). Unlike antibodies with intact Fc effector functions, cadonilimab incorporates an Fc-null design intended to reduce antibody-dependent cell-mediated cytotoxicity (ADCC) and complement-dependent cytotoxicity (CDC)-mediated toxicity (14). Notably, the clinical manifestations of IRRs observed in this study raise the possibility of a CRS-like mechanism, but they are not sufficient to confirm it. This interpretation, however, remains speculative and requires validation through cytokine profiling and mechanistic studies. These convergent mechanisms may provide a potential explanation for the frequent multi-system involvement observed in severe IRRs, which often manifested with CRS-like features including dyspnea, chest tightness, and hemodynamic instability, mirroring the early post-infusion CRS documented in T-cell-redirecting immunotherapy (15).

Importantly, all mechanistic interpretations presented above are hypotheses derived from clinical observations and should be tested in future translational studies incorporating appropriate biospecimen collection (e.g., cytokine profiling and tryptase measurement).

Overall, our findings are broadly consistent with those of Liang et al. (5) regarding the predominant symptom spectrum (cutaneous and respiratory) and the general management approach (corticosteroids plus antihistamines). However, the larger sample size of the present study enabled the detection of patterns not observable in the smaller series (1): severe IRRs were significantly more likely to involve multiple organ systems (2); cardiovascular symptoms were observed exclusively in the severe group (3); patients with prior PD-1 inhibitor exposure were overrepresented in the severe group; and (4) rechallenge outcomes differed by severity grade. Although these observations remain exploratory, they provide new evidence to inform risk stratification and shared decision-making in clinical practice.

### 2 Management of infusion-related reactions: from existing guidelines to clinical practice

Current prescribing information and clinical guidelines (3, 4, 16) recommend temporary infusion interruption and symptomatic therapy (e.g., antihistamines and corticosteroids) with supportive care (oxygen therapy and hemodynamic monitoring) for Grade 1–2 IRRs, with infusion resumed at a reduced rate following symptom resolution. Grade 3–4 IRRs mandate permanent discontinuation.

In addition, guidelines recommend premedication with antipyretics, antihistamines, and corticosteroids administered 30 minutes before infusion to prevent IRRs (16). Dexamethasone-based premedication has been shown to reduce the incidence of IRRs (17), and combination regimens (e.g, diphenhydramine plus dexamethasone) may offer enhanced protection (18). Current practice typically involves intravenous dexamethasone (10–20 mg) with an antihistamine (e.g., diphenhydramine) administered approximately 30 minutes before the initial infusion. In our cohort, 47 patients (90.3%) achieved symptom relief with corticosteroid or antihistamine therapy.

### 3 Challenges and deficiencies in current clinical management practices

Current management practices are primarily based on guidelines developed for monoclonal antibody infusions (4, 19); however, the unique characteristics of bispecific antibodies introduce distinct challenges. First, premedication strategies require tailored adjustments based on patient-specific risk factors, including combination therapy regimens and dose modifications—considerations that remain inadequately addressed by current guidelines. Second, acute-phase management involves not only symptomatic therapy but also critical decisions regarding infusion continuation and rechallenge protocols, which are currently guided largely by individual clinician experience rather than evidence-based consensus (20). Furthermore, clinical teams frequently face resource constraints and time pressures, particularly in centers with high patient volumes.

### 4 Rationale for the proposed preliminary management framework

The proposed framework represents a preliminary synthesis of our institutional experience and the existing literature. It is intended to provide a structured approach for clinical consideration rather than a validated protocol, and requires prospective evaluation. Its key features are as follows: (1) risk-stratified prophylaxis integrating potential risk signals (2); severity-based interventions and (3) structured rechallenge decision-making incorporating multidisciplinary team (MDT) consultation for Grade 3–4 reactions.

### 5 Limitations and future perspectives

This study has several limitations. First, its single-center retrospective design and moderate sample size (n=52) constrained statistical power to detect subtle between-group differences and precluded tumor-specific subgroup analyses. Importantly, because only patients who had already experienced IRRs were included, we could not estimate the overall incidence of cadonilimab-associated IRRs or identify predictors of IRR occurrence. Accordingly, all between-group comparisons should be interpreted as exploratory and hypothesis-generating. Second, the absence of mechanistic biomarkers—including cytokine profiling (21), complement activation markers, and serial tryptase measurements—precludes definitive phenotyping of IRRs (e.g., discriminating CRS from IgE-mediated hypersensitivity), restricting classification to clinical inference. We further acknowledge that tumor-type heterogeneity and the moderate sample size limit the generalizability of our findings and preclude the development of a validated management protocol. Therefore, the proposed management framework should be regarded as preliminary and requires prospective validation in larger, multicenter cohorts.

Future research priorities include: (1) prospective multicenter validation of the proposed framework with iterative refinement (2); translational biomarker discovery through systematic biospecimen collection from IRR cohorts (3); integration of clinical decision support tools within electronic health records and (4) simulation-based team training to ensure proficient protocol implementation during acute events.

## Conclusions

Cadonilimab is associated with distinct IRRs characterized by early onset and multi-system involvement. Through comprehensive analysis of 52 real-world cases, this study delineated the clinical spectrum of these reactions and proposes a preliminary management framework based on our institutional experience. Prospective validation is warranted before this framework can be recommended for routine clinical practice.

## Data Availability

The original contributions presented in the study are included in the article/**Supplementary Material**. Further inquiries can be directed to the corresponding author.
