# Supplementary material for: Clinical characteristics of cadonilimab-associated infusion reactions: a severity-stratified analysis of 52 cases and preliminary management framework
Source: Front Immunol. 2026 Oct 9;17:1815365. doi: 10.3389/fimmu.2026.1815365 (PMC13690811; doi:10.3389/fimmu.2026.1815365)
Supplement: Supplementary file 1 [file Table1.docx]

**Supplementary Table S1 Causality Assessment Details**

| **Patient ID** | **Age** | **Sex** | **Diagnosis** | **Severity grade** | **Combination Regimen** | **Prior PD-1 inhibitor exposure** | **Causality Assessment** |
| --- | --- | --- | --- | --- | --- | --- | --- |
| 001 | 55 | Male | Sigmoid colon cancer | Common | Targeted therapy+ Immunotherapy | Yes | Probable/likely |
| 002 | 55 | Female | Gastric cancer | Severe | Targeted therapy + Immunotherapy | Yes | Probable/likely |
| 003 | 25 | Male | Esophageal squamous cell carcinoma | Common | Chemotherapy + Immunotherapy | No | Probable/likely |
| 004 | 62 | Male | Lung cancer | Common | Monotherapy | Yes | Probable/likely |
| 005 | 62 | Male | Lung cancer | Severe | Targeted therapy+ Immunotherapy | Yes | Certain |
| 006 | 59 | Male | Lung cancer | Severe | Targeted therapy+ Immunotherapy | Yes | Probable/likely |
| 007 | 47 | Male | Lung cancer | Severe | Targeted therapy+ Immunotherapy | Yes | Probable/likely |
| 008 | 33 | Female | Cervical cancer | Severe | Chemotherapy + Immunotherapy | No | Certain |
| 009 | 50 | Female | Cervical cancer | Common | Chemotherapy + Immunotherapy + Targeted therapy | No | Certain |
| 010 | 31 | Female | Cervical cancer | Common | Chemotherapy + Immunotherapy | No | Probable/likely |
| 011 | 65 | Male | Lung cancer | Severe | Targeted therapy+ Immunotherapy | Yes | Certain |
| 012 | 53 | Female | Cervical cancer | Severe | Chemotherapy + Immunotherapy | No | Probable/likely |
| 013 | 76 | Female | Cervical cancer | Common | Chemotherapy + Immunotherapy | No | Possible |
| 014 | 54 | Male | Esophageal squamous cell carcinoma | Severe | Chemotherapy + Immunotherapy + Targeted therapy | Yes | Possible |
| 015 | 58 | Female | Cervical cancer | Severe | Chemotherapy + Immunotherapy + Targeted therapy | No | Probable/likely |
| 016 | 49 | Female | Cervical cancer | Common | Chemotherapy + Immunotherapy | No | Probable/likely |
| 017 | 38 | Male | Renal pelvis carcinoma | Common | Chemotherapy + Immunotherapy | No | Probable/likely |
| 018 | 61 | Male | Nasopharyngeal carcinoma | Common | Chemotherapy + Immunotherapy | No | Probable/likely |
| 019 | 72 | Male | Pancreatic cancer | Common | Chemotherapy + Immunotherapy | No | Probable/likely |
| 020 | 44 | Female | Cervical cancer | Severe | Chemotherapy + Immunotherapy + Targeted therapy | No | Certain |
| 021 | 45 | Female | Cervical cancer | Severe | Chemotherapy + Immunotherapy | No | Probable/likely |
| 022 | 37 | Female | Cervical cancer | Common | Chemotherapy + Immunotherapy | No | Probable/likely |
| 023 | 51 | Female | Cervical cancer | Common | Chemotherapy + Immunotherapy | No | Probable/likely |
| 024 | 53 | Female | Cervical cancer | Severe | Chemotherapy + Immunotherapy + Targeted therapy | No | Probable/likely |
| 025 | 67 | Male | Liver cancer | Common | Monotherapy | No | Probable/likely |
| 026 | 49 | Female | Lung cancer | Severe | Targeted therapy+ Immunotherapy | No | Certain |
| 027 | 58 | Male | Lung cancer | Severe | Targeted therapy+ Immunotherapy | No | Certain |
| 028 | 63 | Female | Lung cancer | Common | Targeted therapy+ Immunotherapy | No | Certain |
| 029 | 67 | Male | Lung cancer | Common | Targeted therapy+ Immunotherapy | No | Possible |
| 030 | 60 | Male | Lung cancer | Common | Targeted therapy+ Immunotherapy | No | Certain |
| 031 | 64 | Male | Liver cancer | Severe | Targeted therapy+ Immunotherapy | No | Probable/likely |
| 032 | 50 | Female | Cervical cancer | Severe | Chemotherapy + Immunotherapy + Targeted therapy | No | Probable/likely |
| 033 | 48 | Female | Ovarian cancer | Common | Chemotherapy + Immunotherapy | No | Certain |
| 034 | 64 | Female | Cervical cancer | Common | Chemotherapy + Immunotherapy | No | Certain |
| 035 | 35 | Male | Gastric cancer | Severe | Chemotherapy + Immunotherapy | Yes | Certain |
| 036 | 62 | Female | Cervical cancer | Common | Chemotherapy + Immunotherapy | Yes | Probable/likely |
| 037 | 52 | Male | Lung cancer | Severe | Monotherapy | No | Certain |
| 038 | 37 | Female | Gastric cancer | Severe | Targeted therapy+ Immunotherapy | Yes | Probable/likely |
| 039 | 39 | Female | Cervical cancer | Severe | Chemotherapy + Immunotherapy | No | Certain |
| 040 | 70 | Female | Endometrial cancer | Common | Chemotherapy + Immunotherapy | No | Probable/likely |
| 041 | 59 | Female | Gastric cancer | Common | Chemotherapy + Immunotherapy | No | Probable/likely |
| 042 | 52 | Male | Lung cancer | Common | Monotherapy | No | Probable/likely |
| 043 | 59 | Female | Cervical cancer | Severe | Chemotherapy + Immunotherapy | Yes | Certain |
| 044 | 61 | Female | Cervical cancer | Common | Chemotherapy + Immunotherapy + Targeted therapy | No | Probable/likely |
| 045 | 58 | Male | Gastric cancer | Common | Chemotherapy + Immunotherapy | No | Probable/likely |
| 046 | 59 | Female | Cervical cancer | Common | Monotherapy | No | Probable/likely |
| 047 | 60 | Female | Cervical cancer | Common | Chemotherapy + Immunotherapy | No | Probable/likely |
| 048 | 59 | Male | Lung cancer | Common | Targeted therapy+ Immunotherapy | No | Probable/likely |
| 049 | 61 | Male | Lung cancer | Common | Targeted therapy+ Immunotherapy | No | Probable/likely |
| 050 | 56 | Male | Gastric cancer | Common | Chemotherapy + Immunotherapy | No | Probable/likely |
| 051 | 62 | Female | Cervical cancer | Common | Chemotherapy + Immunotherapy | Yes | Probable/likely |
| 052 | 57 | Female | Gastric cancer | Common | Chemotherapy + Immunotherapy | No | Probable/likely |
| Notes：  Causality assessment criteria：  The causal relationship between cadonilimab and each IRRs was independently assessed by two clinical pharmacists according to the China Adverse Drug Reaction Reporting and Monitoring Guidelines. Five dimensions were evaluated :  (1) Temporal relationship – IRR onset during or within 24 hours of cadonilimab infusion;  (2) Known reaction pattern – consistency with IRRs described in the cadonilimab prescribing information or published literature;  (3) Dechallenge response – symptom resolution after drug discontinuation or symptomatic treatment;  (4) Rechallenge response – recurrence of similar symptoms upon re-exposure, when applicable;  (5) Exclusion of alternative causes – exclusion of concomitant chemotherapy or targeted agents, infection, tumor progression, and other potential etiologies.  Causality categories：  Certain：All five dimensions met (5/5).  Probable/likely：Four dimensions met; one dimension partially met or not met.  Possible：Three dimensions met; remaining dimensions partially met or not met.  Unlikely：Most dimensions not met.  Unevaluable ：Insufficient information.  Consideration of concomitant agents and alternative causes ：  All patients received cadonilimab as monotherapy or in combination with chemotherapy and/or targeted therapy. For each case, the clinical presentation and time to onset were carefully reviewed to determine whether the event could be attributed to concomitant agents. Events with a reaction pattern consistent with the known IRR profile of cadonilimab and a plausible temporal relationship were attributed to cadonilimab; Cases in which concomitant medications could not be excluded were downgraded accordingly.  Inter-rater agreement ：  Disagreements between the two assessors were resolved through discussion, and consensus was reached for all cases; no cases required adjudication by a third party  Data availability  Dimension-by-dimension scoring records are available from the corresponding author upon reasonable request | | | | | | | |

**Supplementary Table S2 Patient Baseline Characteristics**

| **baseline characteristics** | **N = 52** | **commom,**  **N = 31** | **Severe,**  **N = 21** | **SMD^a^** | **95% CI** | **P-value** |
| --- | --- | --- | --- | --- | --- | --- |
| **age** |  |  |  | 0.54 | -0.03, 1.10 | 0.128^1^ |
| mean ± standard deviation | 54 ± 11 | 57 ± 11 | 51 ± 9 |  |  |  |
| median （Q1，Q3） | 58 (49, 62) | 59 (51, 62) | 53 (45, 58) |  |  |  |
| **Gender, n (%)** |  |  |  | 0.05 | -0.51, 0.60 | 0.871^3^ |
| Male | 23 (44.2%) | 14 (45.2%) | 9 (42.9%) |  |  |  |
| Female | 29 (55.8%) | 17 (54.8%) | 12 (57.1%) |  |  |  |
| **Diagnosis, n (%)** |  |  |  | 0.71 | 0.14, 1.28 | 0.146^2^ |
| Cervical carcinoma | 21 (40.4%) | 12 (38.7%) | 9 (42.9%) |  |  |  |
| Gastric cancer | 7 (13.5%) | 4 (12.9%) | 3 (14.3%) |  |  |  |
| Lung Cancer | 14 (26.9%) | 7 (22.6%) | 7 (33.3%) |  |  |  |
| Carcinoma of sigmoid | 1 (1.9%) | 1 (3.2%) | 0 (0.0%) |  |  |  |
| Esophageal squamous cell carcinoma | 2 (3.8%) | 1 (3.2%) | 1 (4.8%) |  |  |  |
| Renal pelvis carcinoma | 1 (1.9%) | 1 (3.2%) | 0 (0.0%) |  |  |  |
| Nasopharyngeal carcinoma | 1 (1.9%) | 1 (3.2%) | 0 (0.0%) |  |  |  |
| Pancreatic Cancer | 1 (1.9%) | 1 (3.2%) | 0 (0.0%) |  |  |  |
| Liver Cancer | 2 (3.8%) | 1 (3.2%) | 1 (4.8%) |  |  |  |
| Ovarian Cancer | 1 (1.9%) | 1 (3.2%) | 0 (0.0%) |  |  |  |
| Endometrial cancer | 1 (1.9%) | 1 (3.2%) | 0 (0.0%) |  |  |  |
| **Stage, n (%)** |  |  |  | 0.46 | -0.10, 1.02 | 0.423^2^ |
| Stage III | 7 (13.5%) | 5 (16.1%) | 2 (9.5%) |  |  |  |
| Stage IV | 33 (63.5%) | 17 (54.8%) | 16 (76.2%) |  |  |  |
| Recurrence and Metastasis | 12 (23.1%) | 9 (29.0%) | 3 (14.3%) |  |  |  |
| Tumor Metastatic Site**, n (%)** |  |  |  | 0.16 | -0.40, 0.71 | 0.871^3^ |
| ≥3 | 18 (34.6%) | 11 (35.5%) | 7 (33.3%) |  |  |  |
| 2 | 16 (30.8%) | 10 (32.3%) | 6 (28.6%) |  |  |  |
| Oligometastasis | 12 (23.1%) | 7 (22.6%) | 5 (23.8%) |  |  |  |
| Primary Site | 6 (11.5%) | 3 (9.7%) | 3 (14.3%) |  |  |  |
| **PS, n (%)** |  |  |  | 0.53 | -0.03, 1.09 | 0.084¹ |
| 0 | 25 (48.1%) | 14 (45.2%) | 11 (52.4%) |  |  |  |
| 1 | 25 (48.1%) | 17 (54.8%) | 8 (38.1%) |  |  |  |
| 2 | 2 (3.8%) | 0 (0.0%) | 2 (9.5%) |  |  |  |
| **PD-L1 expression, n (%)** |  |  |  | 0.53 | -0.03, 1.10 | 0.722² |
| Negative | 14 (26.9%) | 9 (29.0%) | 5 (23.8%) |  |  |  |
| Positive | 15 (28.8%) | 6 (19.4%) | 9 (42.9%) |  |  |  |
| Unmeasured | 23 (44.2%) | 16 (51.6%) | 7 (33.3%) |  |  |  |
| **Combination regimen, n (%)** |  |  |  | 0.91 | 0.33, 1.49 | 0.026³ |
| Targeted therapy + Immunotherapy | 15 (28.8%) | 6 (19.4%) | 9 (42.9%) |  |  |  |
| Chemotherapy + Immunotherapy | 25 (48.1%) | 19 (61.3%) | 6 (28.6%) |  |  |  |
| Monotherapy | 5 (9.6%) | 4 (12.9%) | 1 (4.8%) |  |  |  |
| Chemotherapy + Immunotherapy + Targeted Therapy | 7 (13.5%) | 2 (6.5%) | 5 (23.8%) |  |  |  |
| **Treatment line, n (%)** |  |  |  | 0.60 | 0.04, 1.17 | 0.191² |
| ˂ Third Line | 42 (80.8%) | 28 (90.3%) | 14 (66.7%) |  |  |  |
| ≥ Third Line | 10 (19.2%) | 3 (9.7%) | 7 (33.3%) |  |  |  |
| **Prior PD-1 inhibitor exposure, n (%)** | 13 (25.0%) | 4 (12.9%) | 9 (42.9%) | 0.71 | 0.14, 1.28 | 0.032² |
| **Prior immune-related adverse events, n (%):** | 7 (13.5%) | 4 (12.9%) | 3 (14.3%) | 0.04 | -0.51, 0.59 | >0.999² |
| **ADR Causality Assessment, n (%)** |  |  |  | 0.72 | 0.15, 1.29 | 0.067² |
| Certain | 15 (28.8%) | 5 (16.1%) | 10 (47.6%) |  |  |  |
| Probable/likely | 34 (65.4%) | 24 (77.4%) | 10 (47.6%) |  |  |  |
| Possible | 3 (5.8%) | 2 (6.5%) | 1 (4.8%) |  |  |  |
| **Onset time(Day)** |  |  |  | 0.07 | -0.49, 0.62 | 0.128¹ |
| Mean±standard deviation | 46 ± 44 | 47 ± 36 | 44 ± 53 |  |  |  |
| median （Q1，Q3） | 33 (22, 54) | 45 (23, 66) | 23 (21, 46) |  |  |  |
| Min, Max | 1, 263 | 1, 179 | 1, 263 |  |  |  |
| **Onset time (min)** |  |  |  | -0.26 | -0.82, 0.30 | 0.722¹ |
| Mean±standard deviation | 59 ± 200 | 36 ± 49 | 93 ± 310 |  |  |  |
| Median （Q1，Q3） | 20 (10, 35) | 20 (7, 35) | 15 (10, 35) |  |  |  |
| Min, Max | 2, 1,440 | 2, 240 | 2, 1,440 |  |  |  |
| **Cadonilimab dose at onset (mg)** |  |  |  | 0.03 | -0.52, 0.59 | 0.716¹ |
| Mean±standard deviation | 188 ± 197 | 191 ± 195 | 184 ± 204 |  |  |  |
| Median （Q1，Q3） | 128 (38, 276) | 160 (34, 268) | 90 (42, 300) |  |  |  |
| Min, Max | 5, 825 | 12, 825 | 5, 708 |  |  |  |
| **Cycle of onset, n (%)** |  |  |  | 0.60 | 0.03, 1.16 | 0.423² |
| C1 | 4 (7.7%) | 3 (9.7%) | 1 (4.8%) |  |  |  |
| C2 | 22 (42.3%) | 11 (35.5%) | 11 (52.4%) |  |  |  |
| C3 | 19 (36.5%) | 12 (38.7%) | 7 (33.3%) |  |  |  |
| C4 | 6 (11.5%) | 5 (16.1%) | 1 (4.8%) |  |  |  |
| C12 | 1 (1.9%) | 0 (0.0%) | 1 (4.8%) |  |  |  |
| a :Standardized Mean Difference; CI = Confidence Interval；¹Wilcoxon rank-sum test;^2^Fisher's exact test；^3^Pearson's Chi-squared test | | | | | | |

**Supplementary Table S3 Clinical Manifestations of IRRs**

| **Organ system** | **N=52** | **Common (N=31)** | **Severe (N=21)** | **P-value¹** |
| --- | --- | --- | --- | --- |
| **Gastrointestinal, n (%)** |  |  |  | 0.365 |
| Abdominal pain | 2 (3.8%) | 2 (6.5%) | 0 (0.0%) |  |
| Abdominal pain and bloating | 2 (3.8%) | 0 (0.0%) | 2 (9.5%) |  |
| Nausea and vomiting | 5 (9.6%) | 3 (9.7%) | 2 (9.5%) |  |
| **Cutaneous, n (%)** |  |  |  | 0.766 |
| Pale lips | 1 (1.9%) | 1 (3.2%) | 0 (0.0%) |  |
| Rash with pruritus | 12 (23.1%) | 7 (22.6%) | 5 (23.8%) |  |
| Rash | 9 (17.3%) | 2 (6.5%) | 7 (33.3%) |  |
| Pruritus | 2 (3.8%) | 2 (6.5%) | 0 (0.0%) |  |
| Perioral swelling | 3 (5.8%) | 2 (6.5%) | 1 (4.8%) |  |
| Periorbital edema | 1 (1.9%) | 1 (3.2%) | 0 (0.0%) |  |
| Facial flushing | 7 (13.5%) | 5 (16.1%) | 2 (9.5%) |  |
| **Cardiovascular, n (%)** |  |  |  | 0.533 |
| Chest tightness ^a^ with palpitations | 3 (5.8%) | 0 (0.0%) | 3 (14.3%) |  |
| Increased heart rate | 2 3.8%) | 0 (0.0%) | 2 (9.5%) |  |
| Decreased blood oxygen | 1 (1.9%) | 0 (0.0%) | 1 (4.8%) |  |
| Palpitations | 3 (5.8%) | 2 (6.5%) | 1 (4.8%) |  |
| Increased blood pressure | 1 (1.9%) | 0 (0.0%) | 1 (4.8%) |  |
| **Respiratory, n (%)** |  |  |  | 0.309 |
| Chest tightness ^a^ with dyspnea | 19 (36.5%) | 7 (22.6%) | 12 (57.1%) |  |
| Dyspnea | 5 (9.6%) | 2 (6.5%) | 3 (14.3%) |  |
| Choking | 2 (3.8%) | 2 (6.5%) | 0 (0.0%) |  |
| Dizziness and fatigue^b^ with dyspnea | 1 (1.9%) | 1 (3.2%) | 0 (0.0%) |  |
| **Neurological, n (%)** |  |  |  | >0.999 |
| Tongue numbness | 1(1.9%) | 1 (3.2%) | 0 (0.0%) |  |
| Numbness in the lips and limbs | 1 (1.9%) | 1 (3.2%) | 0 (0.0%) |  |
| Throat tightness | 3 (5.8%) | 2 (6.5%) | 1 (4.8%) |  |
| Isolated dizziness and fatigue^b^ | 6 (11.5%) | 3 (9.7%) | 3 (14.3%) |  |
| Incontinence | 1 (1.9%) | 0 (0.0%) | 1 (4.8%) |  |
| Hoarseness | 1 (1.9%) | 1 (3.2%) | 0 (0.0%) |  |
| Back pain | 1 (1.9%) | 1 (3.2%) | 0 (0.0%) |  |
| **Systemic, n (%)** |  |  |  | 0.147 |
| Chills and fever | 15 (28.8%) | 8 (25.8%) | 7 (33.3%) |  |
| Confusion | 2 (3.8%) | 0 (0.0%) | 2 (9.5%) |  |
| Fever and night sweats | 4 (7.7%) | 2 (6.5%) | 2 (9.5%) |  |

^1^Fisher's exact test; ^a^ “Chest tightness” was classified under the cardiovascular system when co-occurring with palpitations, and under the respiratory system when co-occurring with dyspnea; ^b^ “Dizziness and fatigue” was classified under the respiratory system when co-occurring with dyspnea, and under the neurological system when present as an isolated symptom.

**Supplementary Table S4 Symptom Distribution by Organ System**

| Organ System | Symptom | Common (n=31) | Severe (n=21) | Total (n=52) |
| --- | --- | --- | --- | --- |
| **Cutaneous** | **Any skin symptom, n(%)** | **20 (64.5%)** | **15 (71.4%)** | **35 (67.3%)** |
|  | Rash with pruritus | 7 (22.6%) | 5 (23.8%) | 12 (23.1%) |
|  | Rash | 2 (6.5%) | 7 (33.3%) | 9 (17.3%) |
|  | Pruritus | 2 (6.5%) | 0 | 2 (3.8%) |
|  | Perioral swelling | 2 (6.5%) | 1 (4.8%) | 3 (5.8%) |
|  | Periorbital edema | 1 (3.2%) | 0 | 1 (1.9%) |
|  | Facial flushing | 5 (16.1%) | 2 (9.5%) | 7 (13.5%) |
|  | Pale lips | 1 (3.2%) | 0 | 1 (1.9%) |
| **Respiratory** | **Any respiratory symptom, n(%)** | **12 (38.7%)** | **15 (71.4%)** | **27 (51.9%)** |
|  | Chest tightness^a^ with dyspnea | 7 (22.6%) | 12 (57.1%) | 19 (36.5%) |
|  | Dyspnea | 2 (6.5%) | 3 (14.3%) | 5 (9.6%) |
|  | Choking | 2 (6.5%) | 0 | 2 (3.8%) |
|  | Dizziness and fatigue^b^ with dyspnea | 1 (3.2%) | 0 | 1 (1.9%) |
| **Cardiovascular** | **Any cardiovascular symptom, n(%)** | **2 (6.5%)** | **8 (38.1%)** | **10 (19.2%)** |
|  | Chest tightness^a^ with palpitations | 0 | 3 (14.3%) | 3 (5.8%) |
|  | Palpitations | 2 (6.5%) | 1 (4.8%) | 3 (5.8%) |
|  | Increased heart rate | 0 | 2 (9.5%) | 2 (3.8%) |
|  | Decreased blood oxygen | 0 | 1 (4.8%) | 1 (1.9%) |
|  | Increased blood pressure | 0 | 1 (4.8%) | 1 (1.9%) |
| **Gastrointestinal** | **Any GI symptom, n(%)** | **5 (16.1%)** | **4 (19.0%)** | **9 (17.3%)** |
|  | Nausea and vomiting | 3 (9.7%) | 2 (9.5%) | 5 (9.6%) |
|  | Abdominal pain | 2 (6.5%) | 0 | 2 (3.8%) |
|  | Abdominal pain and bloating | 0 | 2 (9.5%) | 2 (3.8%) |
| **Neurological** | **Any neurological symptom, n(%)** | **9 (29.0%)** | **5 (23.8%)** | **14 (26.9%)** |
|  | Isolated dizziness and fatigue^b^ | 3 (9.7%) | 3 (14.3%) | 6 (11.5%) |
|  | Throat tightness | 2 (6.5%) | 1 (4.8%) | 3 (5.8%) |
|  | Tongue numbness | 1 (3.2%) | 0 | 1 (1.9%) |
|  | Numbness in the lips and limbs | 1 (3.2%) | 0 | 1 (1.9%) |
|  | Incontinence | 0 | 1 (4.8%) | 1 (1.9%) |
|  | Hoarseness | 1 (3.2%) | 0 | 1 (1.9%) |
|  | Back pain | 1 (3.2%) | 0 | 1 (1.9%) |
| **Systemic** | **Any systemic symptom, n(%)** | **9 (29.0%)** | **10 (47.6%)** | **19 (36.5%)** |
|  | Chills and fever | 8 (25.8%) | 7 (33.3%) | 15 (28.8%) |
|  | Fever and night sweats | 2 (6.5%) | 2 (9.5%) | 4 (7.7%) |
|  | Confusion | 0 | 2 (9.5%) | 2 (3.8%) |

Note: Percentages in the “Total” row were calculated using the total number of patients in each group as the denominator; percentages for individual symptoms were calculated using the same denominator. Patients could experience multiple symptoms within the same organ system; therefore, percentages for individual symptoms may not sum to the "total” row. ^a^ “Chest tightness” was classified under the cardiovascular system when co-occurring with palpitations, and under the respiratory system when co-occurring with dyspnea; ^b^ “Dizziness and fatigue” was classified under the respiratory system when co-occurring with dyspnea, and under the neurological system when present as an isolated symptom.

**Supplementary Table S5 Overlap between Same-Cycle Infusion Resumption and Subsequent-Cycle Re-Exposure**

| Same-cycle infusion resumption outcome | No. of patients (n) | Subsequent-cycle re-exposure (n) | No subsequent-cycle re-exposure (n) | Recurrence after subsequent-cycle re-exposure (n) | No recurrence after subsequent-cycle re-exposure (n) |
| --- | --- | --- | --- | --- | --- |
| Recurrence during same-cycle infusion resumption | 9 | 4 | 5 | 3 | 1 |
| Completed same-cycle infusion resumption without recurrence | 15 | 8 | 7 | 3 | 5 |
| No same-cycle infusion resumption attempted | 28 | 10 | 18 | 8 | 2 |
| **Total** | **52** | **22** | **30** | **14** | **8** |

Note: Subsequent-cycle re-exposure was defined as administration of cadonilimab in a later treatment cycle at a reduced infusion rate. Recurrence rates should be interpreted using the appropriate denominators, as reported in the main text.
